# Dietary biological silage from tambaqui by products affect performance, physiology, and egg quality of young and old laying hens

**DOI:** 10.1007/s11250-026-05084-9

**Published:** 2026-05-28

**Authors:** Cristiane Cunha Guimarães, João Paulo Ferreira Rufino, Alexandre Augusto Barai, Adriene Núzia de Almeida Santos, Maria Fernanda da Silva Gomes, Tiago Cabral Nóbrega, Maiko Willas Soares Ribeiro, Philip Dalbert da Silva Castro, Joana Maia Mendes, Francisco Alberto de Lima Chaves, Joel Lima da Silva Junior, Marco Antonio de Freitas Mendonça, Noedson de Jesus Beltrão Machado, Adriano Teixeira de Oliveira

**Affiliations:** 1 Faculty of Agrarian Sciences, Federal University of Amazonas, Manaus, AM Brazil; 2 Institute of Biological Sciences, Federal University of Amazonas, Manaus, AM Brazil; 3 Institute of Social Sciences, Education, and Animal Science, Federal University of Amazonas, Parintins, AM Brazil; 4 Federal Institute of Education, Science, and Technology of Amazonas - Downtown Campus, Manaus, AM Brazil

**Keywords:** Alternative food, Amazon rainforest, Feed efficiency, *Colossoma macropomum*, Poultry

## Abstract

This study evaluated the effects of increasing inclusion levels of biological silage produced from tambaqui (*Colossoma macropomum*) by-products (BST) in diets of commercial hens at different ages. A completely randomized design in a 2 × 5 factorial arrangement was adopted, comprising two hen ages (23 and 83 weeks) and five BST inclusion levels (0, 1, 2, 3, and 4%), with four replicates of six birds per treatment. Moderate BST inclusion (1–2%) improved egg production, egg mass, and feed efficiency, particularly in younger hens, while maintaining physiological homeostasis. In contrast, higher inclusion levels (≥ 3%) reduced feed intake and performance and negatively affected erythropoietic indicators, with more pronounced effects in older hens, highlighting age-dependent metabolic responses. Egg physical quality, including albumen height, Haugh unit, and eggshell thickness, was optimized at 2% BST, whereas excessive inclusion compromised shell quality and egg weight. Egg composition was modestly affected, mainly in lipid and moisture contents, with interactions between diet and hen age. Sensory evaluation revealed that eggs from hens fed up to 1% BST maintained high acceptability, while higher inclusion levels negatively affected taste. The observed benefits at moderate inclusion levels are likely associated with the presence of highly digestible proteins, bioactive peptides, and fermentation-derived compounds that enhance nutrient utilization and productive efficiency. Conclusively, BST represents a viable and sustainable ingredient for laying hen diets when included at moderate levels (≤ 2%), providing a novel strategy for valorizing aquaculture by-products while optimizing productive performance, physiological responses, and egg quality, considering age-related adaptability.

## Introduction

The prioritization of agricultural products for human consumption has reduced the availability of inputs for animal feeding, increasing feed costs, which account for approximately 70% of total production expenses, and intensifying this challenge in regions with logistical constraints (Cruz et al. 2016; Batalha et al. 2017; Van Zanten et al. 2018; Sandström et al. 2022; Pesti and Choct 2023). In this context, the reuse of agro-industrial by-products has emerged as a strategic alternative, as these residues can partially or totally replace conventional feed ingredients, improve productivity, animal welfare, and product quality, while reducing costs and adding value to nutritionally rich materials (Röös et al. 2017; Van Hal et al. 2019). Additionally, this approach supports sustainability and circular economy strategies by promoting the efficient use of resources and reducing environmental impacts associated with animal production systems (Van Zanten et al. 2018; Van Hal et al. 2019; Sandström et al. 2022; Attia et al. 2025).

Among agro-industrial production chains, aquaculture is one of the largest generators of residues, particularly during fish slaughtering and processing, with by-products such as heads, viscera, scales, skins, and bones often underutilized or improperly discarded, causing environmental, sanitary, and economic impacts (Cândido et al. 2017; Guimarães et al. 2021; Souza Cornélio 2022; Silva et al. 2023; Attia et al. 2025). It is estimated that up to 50–70% of the total biomass processed in fish industries may be converted into waste, representing a substantial annual volume of residues with high potential for valorization (Cândido et al. 2017; Guimarães et al. 2021). In this context, research has increasingly focused on converting these residues into alternative feed ingredients, with fish silage emerging as a promising strategy due to its high nutritional value, especially in protein and lipids (Batalha et al. 2018; Assunção et al. 2019; Ferreira et al. 2021; Guimarães et al. 2021; Silva et al. 2023; Puja et al. 2024). This process involves pH reduction of the raw material either by the addition of organic acids (chemical silage) or by microbial fermentation that promotes autolytic enzymatic activity (biological silage), enabling the efficient transformation of fish by-products into a stable and nutritionally valuable feed resource (Batalha et al. 2017, 2018; Guimarães et al. 2019, 2021). Within this scenario, tambaqui (*Colossoma macropomum*), the main native species produced by Brazilian aquaculture and also widely cultivated in China (Cyrino et al. 2010; Alhazzaa et al. 2019; FAO 2020; Gervaz et al. 2023), contributes substantially to residue generation along its processing chain (Ferreira et al. 2021; Souza Cornélio 2022), and these by-products, originating from one of the fastest-growing sectors of animal production worldwide (FAO 2022), represent a valuable resource with high potential for reuse (Boscolo et al. 2011; Ferreira et al. 2021; Souza Cornélio 2022).

However, the effective utilization of alternative feed ingredients such as fish residues depends not only on their nutritional composition but also on the physiological capacity of the animals to digest and absorb nutrients, which is influenced by age (Kulshreshtha et al. 2024; Şekeroğlu et al. 2024). In older laying hens, digestive efficiency tends to decline due to reduced secretion of digestive enzymes, such as proteases and lipases, impairing nutrient breakdown (Muir et al. 2022; Tainika et al. 2024). Additionally, structural changes in the intestinal mucosa, including reduced villus height and absorptive surface area, can limit nutrient absorption (Muir et al. 2022; Kulshreshtha et al. 2024). These age-related physiological constraints may reduce the capacity of older hens to efficiently utilize alternative ingredients such as fish silage, potentially leading to differences in productive responses when compared with younger birds (Muir et al. 2022; Şekeroğlu et al. 2024; Tainika et al. 2024).

Accordingly, the hypothesis of this study is based on the potential of biological silage from tambaqui by-products (BST) to serve as a viable alternative feed ingredient for inclusion in diets of commercial hens at different ages, given that age has been shown to influence the metabolic utilization of feed ingredients and their nutrients (Gu et al. 2021; Yamada et al. 2021). Previous studies evaluating the effects of fish by-product silage on the digestibility of commercial hens (Batalha et al. 2017; Guimarães et al. 2019, 2021), as well as on productive performance and egg quality (Batalha et al. 2018; Guimarães et al. 2025), have reported positive outcomes, including improvements in egg production, feed efficiency, and egg quality traits, as well as favorable changes in lipid metabolism, demonstrating good economic feasibility and contributing to a circular economy perspective within the poultry industry (Oliveira 2018; Batalha et al. 2019). However, these studies have primarily evaluated responses under single age conditions, without addressing whether age-dependent physiological differences modulate the efficacy and safety of fish silage inclusion in laying hen diets. Therefore, a critical knowledge gap remains regarding how the interaction between hen age and BST inclusion level influences productive performance, physiological responses, and egg quality (Batalha et al. 2018, 2019; Guimarães et al. 2021, 2025).

In this context, the present study advances beyond previous research by explicitly investigating the interaction between two distinct physiological stages (young and old hens) and graded levels of BST inclusion, allowing the identification of age-specific response thresholds and potential metabolic limitations. We hypothesize that the response to BST is not uniform across ages, and that older hens exhibit reduced tolerance to higher inclusion levels due to limitations in digestive efficiency, nutrient absorption, and metabolic adaptability. Based on this framework, the present study was conducted to investigate the effects of increasing levels of BST in diets for commercial hens with different ages (23 and 83 weeks) on productive performance, physiological parameters (hematological and plasma biochemical, with particular emphasis on indicators of metabolic status, physiological homeostasis, and overall animal health), as well as the physical, chemical, and sensory quality of eggs, providing insights into how this alternative feed ingredient not only supports productivity but also safeguards the health and physiological well-being of laying hens at different production stages.

## Materials and methods

This study was conducted at the Research Poultry Farm of the Federal University of Amazonas (UFAM), located on the university campus in Manaus, Amazonas, Brazil. All experimental procedures were reviewed and approved by the Animal Ethics Committee of UFAM (protocol no. 005/2022) and were carried out in full compliance with internationally accepted ethical standards for the use and care of animals.

### Processing of tambaqui residues and silage production

Residues consisting of viscera, gills, scales, and fins generated during the industrial processing of tambaqui (800-1,300 g) were obtained from a commercial facility in Manaus, Amazonas, Brazil. The material was transported under refrigerated conditions, stored frozen, and subsequently thawed and subjected to thermal treatment at 65 °C for 5 min as a mild pre-treatment aimed at reducing the initial microbial load and facilitating subsequent fermentation, without compromising the activity of endogenous autolytic enzymes or the viability of lactic acid bacteria added during the silage process. After mechanical processing, the biomass was transferred to containers for silage production.

For silage preparation, the processed biomass was mixed with cassava trimmings (10% w/w) based on the total weight of the silage mixture, as a carbohydrate source, an inoculum of *Lactobacillus plantarum* (2.5% v/w) relative to the total mixture, benzoic acid (0.1% w/w), and ascorbic acid (0.1% w/w), following the methodology described by Vidotti et al. (2003, 2011). The inclusion of benzoic acid at 0.1% was intended to inhibit undesirable microbial growth, while ascorbic acid at the same concentration acted as an antioxidant to limit lipid oxidation and preserve silage quality during fermentation and storage. The mixture was homogenized, stored under anaerobic conditions, and fermented at room temperature for 14 days, with periodic monitoring of pH, titratable acidity, and organoleptic characteristics. These parameters were evaluated at the beginning of the process (day 0), during fermentation (days 3 and 7), and at the end of the fermentation period (day 14), allowing the assessment of fermentation dynamics and stabilization.

After the fermentation period, the silage was spread onto aluminum trays and dried in a forced-air oven at 65 °C (149 °F) for 72 h to reduce moisture content. This temperature is commonly used in fish by-product processing as it allows effective moisture removal while minimizing lipid oxidation and preserving the nutritional quality of lipids. Once dried, proximate composition analyses were conducted to determine moisture, mineral matter, lipid, fiber, and soluble carbohydrate contents of the BST, following AOAC (2019) methodologies. Microbiological analyses were also performed in accordance with the standards of the Brazilian Ministry of Agriculture, Livestock, and Supply (2018), including assessments of total and thermotolerant coliforms, *Salmonella* spp., *Staphylococcus* spp., molds, and yeasts. After confirmation of physicochemical and microbiological quality, the processed silage was forwarded to the poultry research sector for use in the experimental trial with commercial hens.

### Facilities, animals, diets and experimental design

The aviary measured 17 m in length, 3.5 m in width, and had a ceiling height of 3.25 m, incorporating structural adaptations aimed at promoting bird welfare. Ambient temperature and relative humidity were continuously monitored using a digital thermohygrometer, with mean values of 28.3 °C (82.94 °F) and 62.11%, respectively. Throughout the experimental period, the hens were closely monitored for signs of thermal stress associated with environmental conditions; however, no indications of heat or cold stress were observed during the study.

The experimental period lasted 63 days and was divided into three subperiods of 21 days each, to allow repeated measurements over time and increase data reliability without changes in experimental conditions. A total of 240 commercial hens of the Hisex Brown strain were used, comprising 120 younger hens (23 weeks of age) and 120 older hens (83 weeks of age), following a seven-day adaptation period to the experimental diets and housing conditions. At the beginning of the trial, birds were weighed to standardize the experimental groups, with mean body weights of 1.87 ± 0.18 kg for younger hens and 1.93 ± 0.13 kg for older hens. The hens were housed in galvanized wire cages measuring 0.45 m in height, 0.40 m in width, and 1.00 m in length, with six birds per cage arranged in a single row. Each cage was equipped with a trough feeder and nipple drinkers to allow ad libitum access to feed and water. A lighting program of 16 h of light per day was adopted, consisting of 12 h of natural light supplemented with 4 h of artificial light, and maintained throughout the experimental period. Eggs were collected twice daily (09:00 and 15:00 h), and all daily records, including egg production and mortality, were systematically registered.

The hens were allocated in a completely randomized design arranged in a 2 × 5 factorial scheme, comprising two evaluated factors: hen age (23 and 83 weeks) and levels of BST inclusion in the diets (0, 1, 2, 3, and 4%). Each dietary treatment consisted of four replicates with six birds per replicate, totaling 24 hens per treatment. The replicate (i.e., the group of six hens housed in each cage) was considered the experimental unit for all statistical analyses. The number of replicates was defined based on experimental designs commonly used in poultry nutrition studies, in which four replicates per treatment are considered adequate to detect biologically relevant differences under controlled conditions and relatively low experimental variability (Sakomura and Rostagno 2016). The experimental diets (Table 1) were formulated to meet the nutritional requirements of commercial laying hens at different ages based on the reference values provided by Rostagno et al. (2024), complemented by specific guidelines for the Hisex Brown strain (Hendrix and Genetics 2021), as well as the previously determined composition of BST. To ensure accuracy, proximate analyses of the diets were performed to verify and confirm the calculated nutritional values.

**Table 1:** Composition of experimental diets containing biological silage from tambaqui by-products (BST)

Feedstuffs	Younger hens (23 weeks)					Older hens (83 weeks)				
Corn (7.88%)^1^	63.63	63.29	62.95	62.61	62.27	65.80	65.30	64.42	64.29	63.79
Soybean meal (46%)^1^	24.05	23.37	22.69	22.01	21.33	21.41	20.76	20.11	19.46	18.81
BST level	0.00	1.00	2.00	3.00	4.00	0.00	1.00	2.00	3.00	4.00
Limestone	9.32	9.32	9.32	9.32	9.32	9.78	9.91	10.42	10.17	10.30
Dicalcium phosphate	1.91	1.91	1.92	1.93	1.94	1.93	1.94	1.95	1.95	1.96
Vit. min. supplement^2^	0.60	0.60	0.60	0.60	0.60	0.60	0.60	0.60	0.60	0.60
Salt	0.33	0.34	0.34	0.34	0.34	0.29	0.29	0.30	0.30	0.30
DL-methionine (99%)^3^	0.16	0.17	0.18	0.19	0.20	0.19	0.20	0.21	0.22	0.23
Total	100.00	100.00	100.00	100.00	100.00	100.00	100.00	100.00	100.00	100.00
Nutrient	Nutritional levels^4^									
M.E., kcal.kg^− 1^	2,850.00	2,850.00	2,850.00	2,850.00	2,850.00	2,900.00	2,900.00	2,900.00	2,900.00	2,900.00
Crude protein, %	16.50	16.50	16.50	16.50	16.50	15.50	15.50	15.50	15.50	15.50
Calcium, %	4.15	4.15	4.15	4.15	4.15	4.32	4.32	4.32	4.32	4.32
Available phosphorus, %	0.45	0.45	0.45	0.45	0.45	0.45	0.45	0.45	0.45	0.45
Fats, %	2.53	2.84	3.19	3.47	3.79	2.57	2.88	3.24	3.53	3.87
Crude fiber, %	2.66	2.63	2.59	2.56	2.53	2.55	2.52	2.48	2.45	2.41
NDF, %	10.66	10.53	10.39	10.26	10.12	10.54	10.39	10.24	10.09	9.94
ADF, %	4.04	3.98	3.92	3.85	3.79	3.92	3.85	3.78	3.71	3.64
Methionine + Cystine, %	0.70	0.70	0.70	0.70	0.70	0.70	0.70	0.70	0.70	0.70
Methionine, %	0.42	0.42	0.43	0.43	0.43	0.43	0.44	0.45	0.45	0.46
Lysine, %	0.82	0.80	0.78	0.76	0.74	0.76	0.74	0.72	0.70	0.68
Threonine, %	0.63	0.62	0.61	0.59	0.58	0.60	0.58	0.57	0.56	0.54
Tryptophan, %	0.19	0.18	0.18	0.18	0.17	0.18	0.17	0.17	0.16	0.16
Sodium, %	0.17	0.17	0.17	0.17	0.17	0.15	0.15	0.15	0.15	0.15

^1^ Values in parentheses indicate the protein content of these feedstuffs

^2^ Guaranteed levels per kilogram of the product: Vitamin A 2,000,000 IU, Vitamin D3 400,000 IU, Vitamin E 2,400 mg, Vitamin K3 400 mg, Vitamin B1 100 mg, Vitamin B2 760 mg, Vitamin B6 100 mg, Vitamin B12 2,400 mcg, Niacin 5,000 mg, Calcium Pantothenate 2,000 mg, Folic acid 50 mg, Coccidiostat 12,000 mg, Choline 50,000 mg, Copper 1,200 mg, Iron 6,000 mg, Manganese 14,000 mg, Zinc 10,000 mg, Iodine 100 mg. Selenium 40 mg. Vehicle q.s.p. 1,000 g

^3^ The value in parentheses indicates the purity of the amino acid

^4^ Levels analyzed and calculated on a dry matter basis

### Productive performance

The productive performance of the hens was evaluated according to the methodology described by Rufino et al. (2021). Performance variables were recorded at seven-day intervals and included feed intake (g/bird/day), egg production (%), feed conversion ratio expressed as kilograms of feed per kilogram of eggs produced (kg/kg), feed conversion ratio expressed as kilograms of feed per dozen eggs produced (kg/dz), and egg mass (g) per replicate. Feed intake was calculated as the difference between feed offered and refusals collected and weighed at the end of each period; egg production was determined as the percentage of eggs produced per replicate per day; egg mass was obtained by multiplying egg production by average egg weight; and feed conversion ratios were calculated based on feed intake relative to egg mass and dozen eggs produced in each subperiod. During the final two days of each 21-day subperiod, eggs from each replicate were randomly collected and used for egg quality analyses.

### Hematological and plasma biochemical parameters

Eight hens per treatment (2 per replicate) were randomly selected for blood collection and analysis of hematological and plasma biochemical parameters. Blood sampling was performed in the morning (between 08:00 and 10:00 h), after a standardized interval following feed access, in order to minimize short-term postprandial variation in biochemical parameters. Blood sampling was performed once, at the end of the each 21-days subperiods, in order to reflect the cumulative physiological effects of the dietary treatments. One milliliter of blood was collected directly from the ulnar vein using disposable syringes containing heparin as an anticoagulant (5,000 IU per sample).

For hematological analyses, the collected blood was used to determine circulating erythrocyte counts (M/mm³) in a Neubauer chamber after dilution in formaldehyde-citrate solution and visualization under an optical microscope (Nikon Eclipse E-50i, DM3000, Tokyo, Japan) with a 40× objective lens. Hemoglobin concentration (g/dL) was determined using the Drabkin reagent method with spectrophotometric reading (model K37-UVVIS, Kasvi©, São José dos Pinhais, Brazil) (Thakkar et al. 2021), while hematocrit (%) was determined by the microhematocrit method, with centrifugation of heparinized microcapillary tubes at 10,000 rpm for 5 min (Goldenfarb et al. 1971). Based on these results, mean corpuscular volume (MCV, µm³), mean corpuscular hemoglobin (MCH, pg/cell), and mean corpuscular hemoglobin concentration (MCHC, g/dL) were calculated according to Tavares-Dias and Moraes (2004).

After hematological analyses, the blood samples were immediately centrifuged at 5,000 rpm for 5 min to separate the plasma for biochemical analyses. This centrifugation protocol was adopted based on standard procedures for plasma separation in avian blood samples (Thrall 2012). The plasma samples were properly identified and stored in an ultrafreezer at -80 °C until laboratory evaluation. The same plasma samples obtained from each bird were used for all biochemical determinations.

For plasma biochemical analyses, samples were subjected to commercial enzymatic-colorimetric assay kits according to the manufacturer’s instructions, and absorbance readings were obtained using a spectrophotometer (model K37-UVVIS, Kasvi©, São José dos Pinhais, Brazil) at specific wavelengths for each assay. Commercial kits were obtained from InVitro Diagnóstica (Itabira, MG, Brazil), including glucose, triglycerides, cholesterol, total protein, albumin, and uric acid assays, all performed according to the manufacturer’s specifications.

### Physical quality of the eggs

For physical egg quality analysis, the study adhered to the methodology described by Rufino et al. (2021). At the end of the each 21-days subperiods, five eggs per treatment were stored for one hour at room temperature and then weighed using an electronic balance with a precision of 0.01 g. They were placed in wire baskets and immersed in buckets containing sodium chloride (NaCl) solutions with densities ranging from 1.075 to 1.100 g/cm³, in intervals of 0.005, to determine specific gravity. Subsequently, eggs were placed on a flat glass plate to measure albumen height, yolk height, and yolk diameter using an electronic caliper. Albumen and yolk were separated using a manual separator, then transferred to individual plastic cups and weighed on an analytical balance. Eggshells were washed, dried in an oven at 50 °C (122 °F) for 48 h, and weighed. Eggshell thickness (mm) was measured using a digital caliper after removing shell membranes. Measurements were taken at three different regions of the dried eggshell: broad end, equator, and sharp end, and the average of these measurements was used for statistical analysis. Yolk color was evaluated using a ROCHE© colorimetric fan (with a scale ranging from 1 to 15). All yolk color evaluations were performed by a single trained evaluator under standardized lighting conditions to ensure consistency and minimize subjective variation. The Haugh unit was calculated using the formula (1) (Haugh 1937):

1 $$H_{unit}=100*\log(H+7.57-1.7*W^{0.37})$$

Where:

H = albumen height (mm)

W = egg weight (g)

### Chemical composition and lipid oxidation of the yolk

At the end of the each 21-days subperiods, eight eggs from each treatment were subjected to chemical composition analysis, which included the evaluation of moisture (%), minerals (%), fats (%), and proteins (%). These analyses were performed following the methods described by the Association of Official Analytical Chemists (AOAC 2019).

### Sensory characteristics of the eggs

For sensory analysis, one egg from each replicate per treatment was selected at the end of the experimental period, in addition to a fifth egg randomly chosen among the replicates, totaling five eggs per treatment. A total of 40 untrained judges of both genders were randomly selected to evaluate the sensory attributes of the eggs, including appearance, acidity, aroma, color, and taste. Eggs were cooked in boiling water for 10 min, cooled to room temperature, peeled, and longitudinally cut into two halves, with each half subsequently divided into four portions, yielding eight sensory samples per egg. Thus, each treatment generated 40 individual portions (5 eggs × 8 portions), allowing one portion per treatment to be served to each judge. Sensory evaluation was conducted using a nine-point hedonic scale, ranging from 1 (“disliked extremely”) to 9 (“liked extremely”), in accordance with the methodology described by Hayat et al. (2010) and Berkhoff et al. (2020).

### Statistical analyses

The adopted statistical model (2) was as follows, based on the premises described by Freitas (2022):

2 $$Y_{ijk}=\upmu+\mathrm{A}_i+\mathrm{B}_j+(\mathrm{A}*\mathrm{B})_{ij}+\epsilon_{ijk}$$

where:

Y_ijk_ = Observed value for the variable under study

µ = Overall mean

A_i_​ = effect of hen age

B_j_​ = effect of BST inclusion level

(A * B)_ij_​ = interaction between hen age and BST level

ϵ_ik_ = Experimental error.

The replicate (i.e., the cage containing six hens) was considered the experimental unit for all statistical analyses, and all measured variables were averaged per replicate prior to analysis. All data were analyzed by two-way ANOVA using the R software (2021), following the guidelines outlined by Logan (2010). The assumptions of normality and homogeneity of variances were evaluated using the Shapiro-Wilk and Levene tests, respectively. When significant effects were detected, Tukey’s honestly significant difference (HSD) test was applied for multiple comparisons among means. The results are presented as means, and statistical significance was declared at *p* ≤ 0.05.

## Results

### Physicochemical and microbiological quality of BST

During the silage production, the color of the ensiled mass evolved from brown to reddish-pink, reaching dark brown by the 14th day. The strong fish odor diminished, replaced by a mildly acidic or fruity aroma. Liquefaction occurred within the first 72 h, resulting in a semi-pasty consistency that persisted throughout the process. The pH dropped from 6.47 on day 0 to 5.93 on day 3, stabilizing around 5.95 by day 14. Titratable acidity increased from 2.13% to 10.13% (expressed as lactic acid equivalents), reflecting effective fermentation. Microbiological analysis showed low levels of coliforms, molds, and yeasts, with no detection of *E. coli*, *Salmonella spp.*, or *Staphylococcus aureus*, confirming the safety of BST for poultry diets. Proximate composition analysis (Table 2) revealed enhanced nutrient concentration after drying, with increased dry matter, protein, fat, and mineral content compared to the raw residues.

**Table 2:** Proximate composition of fresh residual mass and biological silage from tambaqui by-products

Proximate composition	Fresh waste*	Biological silage*
Dry matter, %	44.61 ± 2.87	87.69 ± 0.17
Crude protein, %	22.67 ± 1.81	44.98 ± 0.59
Fats, %	7.38 ± 0.35	17.89 ± 0.47
Minerals, %	11.70 ± 1.15	21.87 ± 0.69
Crude fiber, %	1.62 ± 0.28	2.44 ± 1.24
Soluble carbohydrates, %	1.23 ± 0.32	0.50 ± 1.06

*All nutrient values were calculated on a dry matter basis. Mean ± standard deviation of three replicates

### Productive performance

**Fig. 1:**
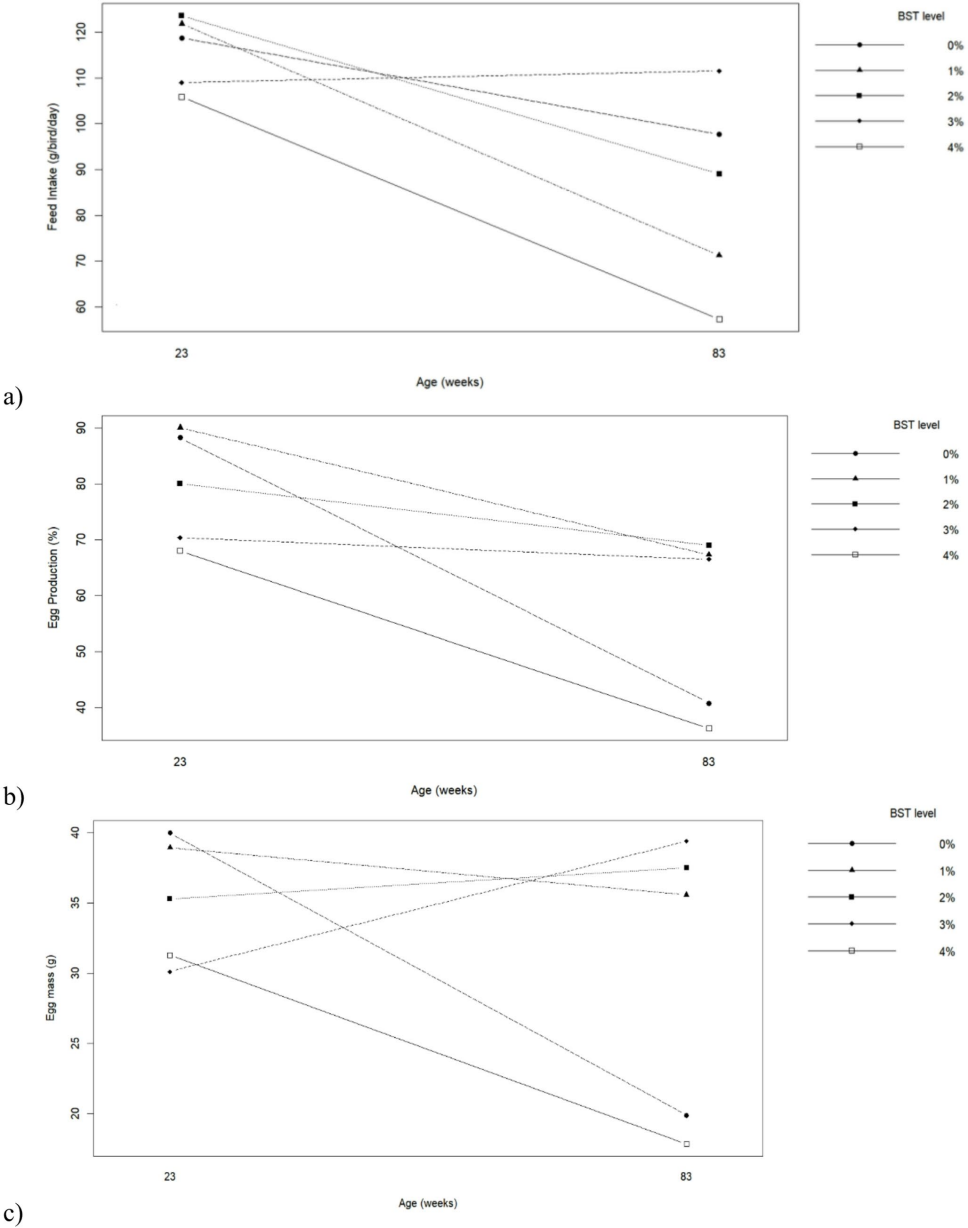
Interaction between biological silage from tambaqui by-products levels in the diets and hen’s age on (**a**) Feed intake, (**b**) Egg production and (**c**) Egg mass of commercial hens

The productive performance of commercial hens was significantly influenced by hen age, BST inclusion level, and their interaction for most evaluated variables (Table 3; Fig. 1). Younger hens (23 weeks) showed higher feed intake, egg production, and egg mass, as well as better feed efficiency expressed both as kg/kg and kg/dz, compared with older hens (83 weeks) (*p* ≤ 0.05). Regarding BST inclusion, diets containing 1% and 2% BST resulted in higher egg production and egg mass and improved feed efficiency compared with the control diet (0% BST) (*p* ≤ 0.05). The best overall performance was observed at 1% BST, with the lowest feed conversion ratios and the highest egg mass values. In contrast, inclusion levels of 3% and especially 4% BST negatively affected performance, with significant reductions in feed intake, egg production, and egg mass (*p* ≤ 0.05). A significant interaction between hen age and BST level was observed for feed intake, egg production, and egg mass (*p* ≤ 0.05). As illustrated in Fig. 1, younger hens maintained higher productive responses at moderate BST inclusion levels (1–2%), whereas older hens exhibited a more pronounced decline in performance when BST levels exceeded 2%, particularly at 4% inclusion.

**Table 3:** Performance of commercial hens with different ages fed diets containing increasing levels of biological silage from tambaqui by-products

Factors^1^	Variables^2^				
	FI	EP	FEKG	FEDZ	EM
Age					
23 weeks	115.78 ± 8.70^a^	79.34 ± 13.26^a^	2.62 ± 0.22^b^	1.77 ± 0.28^b^	35.12 ± 6.05^a^
83 weeks	85.35 ± 9.64^b^	55.95 ± 21.67^b^	3.40 ± 0.82^a^	2.12 ± 0.31^a^	30.03 ± 6.26^b^
BST					
0	108.16 ± 11.93^ab^	64.49 ± 25.87^b^	3.85 ± 1.53^a^	2.28 ± 0.83^a^	29.92 ± 11.06^b^
1	106.53 ± 27.13^b^	78.63 ± 17.96^a^	2.49 ± 0.60^c^	1.49 ± 0.32^c^	37.26 ± 7.41^a^
2	106.33 ± 18.82^b^	74.52 ± 11.63^a^	2.62 ± 0.37^bc^	1.72 ± 0.25^bc^	36.40 ± 4.83^a^
3	110.21 ± 12.91^a^	68.44 ± 22.13^ab^	3.25 ± 2.25^ab^	2.31 ± 0.45^a^	34.75 ± 13,87^a^
4	81.59 ± 26.35^c^	52.12 ± 20.90^c^	2.84 ± 0.83^b^	1.95 ± 0.44^b^	24.55 ± 9.57^b^
Effect	p-value				
Age^3^	< 0.01	< 0.01	0.05	0.05	0.02
BST^3^	< 0.01	< 0.01	0.04	0.05	0.05
Interaction^4^	< 0.01	< 0.01	0.06	0.16	< 0.01
CV^5^	11.38	11.52	14.60	12.14	12.21

^1^ Age = Age of the hens at the beginning of the study. BST = Level of biological silage from tambaqui by-products in the diets

^2^ FI = Feed intake (g/bird/day). EP = Egg production (%). FEKG = Feed efficiency (kg/kg). FE = Feed efficiency (kg/dz). EM = Egg mass (g)

^3^ Means followed by lowercase letters in the column indicate a significant effect of the evaluated factor on the analyzed variable according to Tukey’s test at 0.05

^4^ A p-value above 0.05 demonstrates the direct influence of one factor on the result of the other and vice versa

^5^ CV = Coefficient of variation

### Hematological parameters

**Fig. 2:**
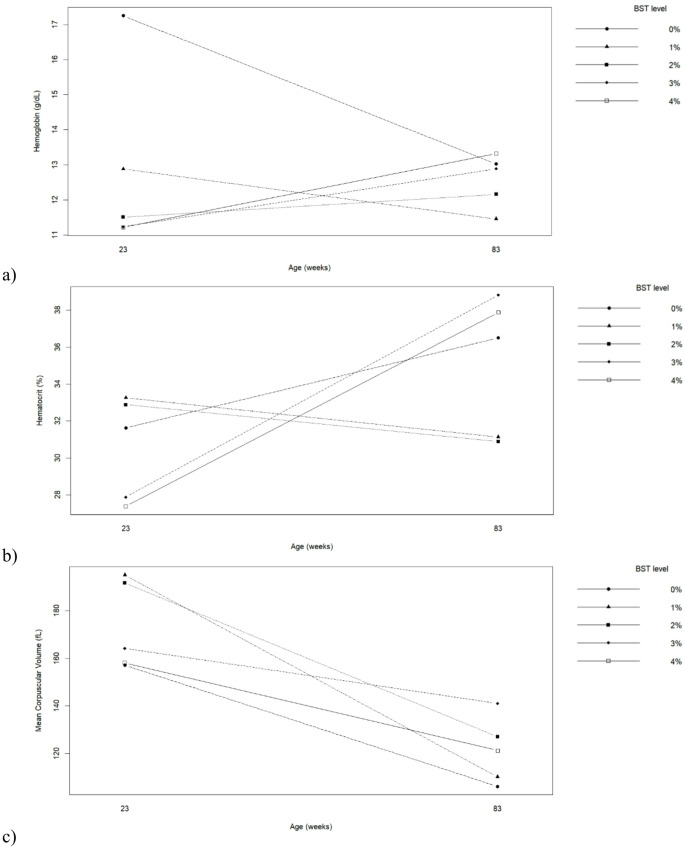

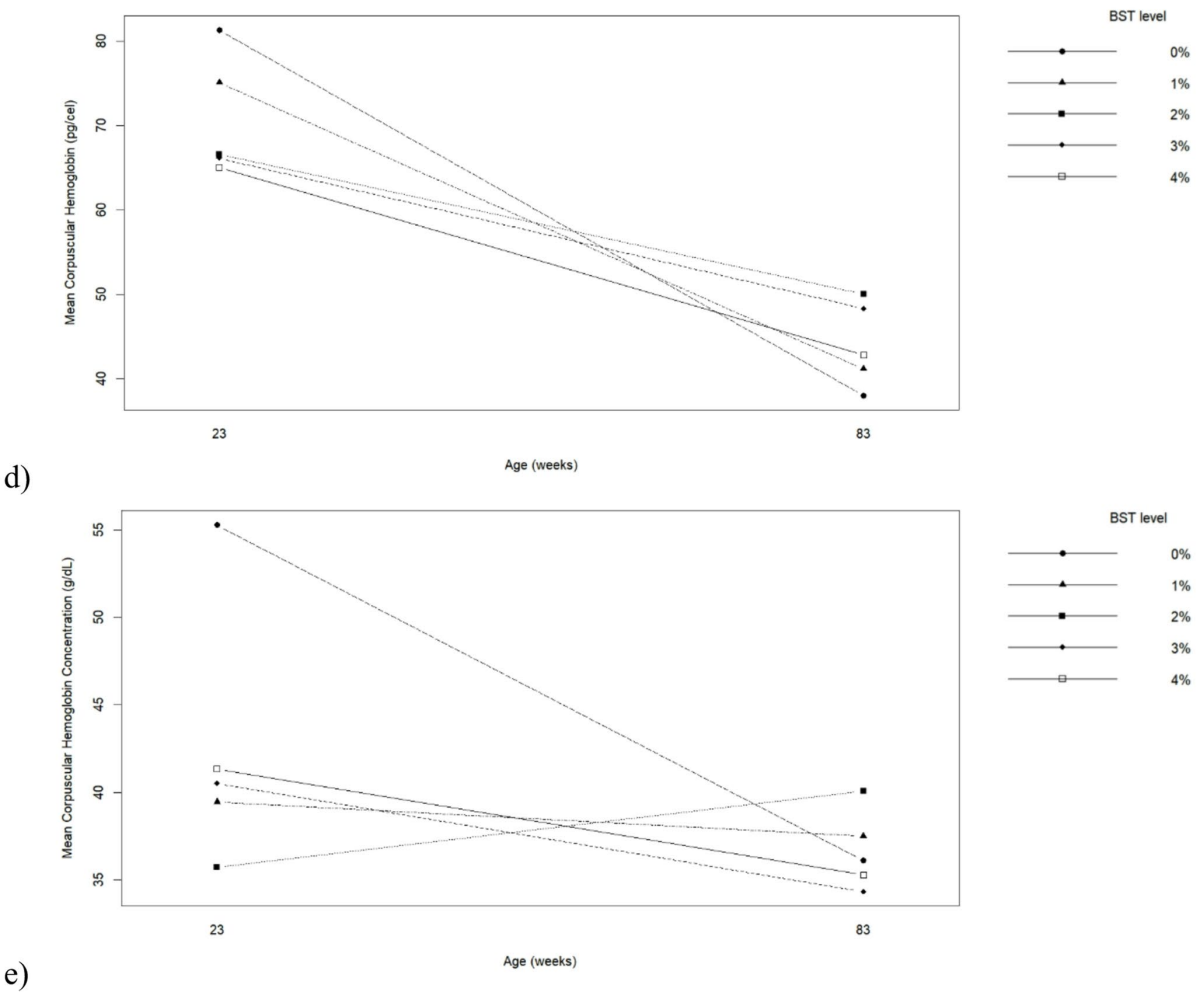
Interaction between biological silage from tambaqui by-products levels in the diets and hen’s age on (**a**) Hemoglobin, (**b**) Hematocrit, (**c**) Mean Corpuscular Volume (**d**) Mean Corpuscular Hemoglobin and (**e**) Mean Corpuscular Hemoglobin Concentration of commercial hens

Hematological parameters were affected by hen age, BST inclusion level, and their interaction (Table 4; Fig. 2). Older hens showed higher red blood cell counts and hematocrit values, whereas younger hens presented higher mean corpuscular volume, mean corpuscular hemoglobin, and mean corpuscular hemoglobin concentration (*p* ≤ 0.05). BST inclusion significantly influenced hemoglobin concentration, erythrocyte count, hematocrit, MCV, and MCHC (*p* ≤ 0.05). Birds fed the control diet (0% BST) presented higher hemoglobin concentration and erythrocyte counts compared to hens receiving diets containing BST, particularly at higher inclusion levels (≥ 3%). Moderate BST inclusion (1–2%) maintained hematological parameters within physiological ranges, whereas higher inclusion levels (3–4%) led to reductions in hemoglobin concentration and erythrocyte count, indicating a potential adverse effect on erythropoietic status. Significant interactions between hen age and BST inclusion were detected for hemoglobin, hematocrit, MCV, MCH, and MCHC (*p* ≤ 0.05). Figure 2 demonstrates that younger hens were more resilient to BST inclusion up to 2%, while older hens showed more pronounced hematological alterations at higher inclusion levels.

**Table 4:** Hematological parameters of commercial hens with different ages fed diets containing increasing levels of biological silage from tambaqui by-products

Factors^1^	Variables^2^					
	HEM	RBC	HET	MCV	MCH	MCHC
Age						
23 weeks	12.82 ± 4.07	1.84 ± 0.50^b^	30.60 ± 5.58^b^	173.12 ± 46.28^a^	70.85 ± 21.86^a^	42.45 ± 13.64^a^
83 weeks	12.57 ± 2.46	2.95 ± 0.55^a^	35.03 ± 5.66^a^	121.17 ± 22.13^b^	44.09 ± 12.22^b^	36.65 ± 8.94^b^
BST						
0	14.43 ± 4.84^a^	3.09 ± 0.92^a^	34.87 ± 4.94^a^	123.17 ± 43.48^c^	52.46 ± 31.15^b^	42.48 ± 17.75^a^
1	11.93 ± 2.56^c^	2.49 ± 0.64^b^	31.83 ± 5.66^b^	138.44 ± 55.55^b^	52.48 ± 22.58^b^	38.14 ± 8.97^b^
2	11.94 ± 1.97^c^	2.24 ± 0.48^b^	31.54 ± 3.83^b^	148.53 ± 45.37^a^	55.57 ± 14.77^a^	38.61 ± 9.26^b^
3	12.33 ± 2.40^b^	2.41 ± 0.64^b^	35.16 ± 8.26^a^	148.73 ± 20.40^a^	54.28 ± 15.70^a^	36.39 ± 9.09^b^
4	12.61 ± 2.19^b^	2.67 ± 0.74^b^	34.37 ± 5.80^a^	133.56 ± 22.53^b^	50.24 ± 13.81^c^	37.30 ± 6.54^b^
Effect	p-value					
Age^3^	0.65	< 0.01	0.01	< 0.01	< 0.01	< 0.01
BST^3^	0.01	< 0.01	< 0.01	0.01	0.80	0.05
Interaction^4^	< 0.01	0.22	< 0.01	0.01	0.02	< 0.01
CV^5^	14.33	9.08	7.86	9.17	8.48	8.59

^1^ Age = Age of the hens at the beginning of the study. BST = Level of biological silage from tambaqui by-products in the diets

^2^ HEM = Hemoglobin (g/dL). RBC = Red Blood Count (M/mm^3^). HET = Hematocrit (%). MCV = Mean Corpuscular Volume (fL). MCH = Mean Corpuscular Hemoglobin (pg/cel). MCHC = Mean Corpuscular Hemoglobin Concentration (g/dL)

^3^ Means followed by lowercase letters in the column indicate a significant effect of the evaluated factor on the analyzed variable according to Tukey’s test at 0.05

^4^ A p-value above 0.05 demonstrates the direct influence of one factor on the result of the other and vice versa

^5^ CV = Coefficient of variation

### Plasma biochemical parameters

Plasma biochemical parameters were significantly influenced by hen age (Table 5). Older hens exhibited higher concentrations of total proteins, triglycerides, cholesterol, and albumin, whereas younger hens showed higher plasma glucose and uric acid levels (*p* ≤ 0.05). BST inclusion affected total protein, cholesterol, and albumin concentrations (*p* ≤ 0.05). Diets containing 1% and 2% BST resulted in increased plasma protein levels compared with the control diet, while cholesterol levels decreased progressively as BST inclusion increased, with the lowest values observed at 3% and 4% BST. Albumin concentration increased at higher BST levels (3–4%). No significant interaction between hen age and BST level was observed for plasma biochemical parameters (*p* > 0.05), indicating that age and diet exerted independent effects on these variables.

**Table 5:** Plasma biochemical parameters of commercial hens with different ages fed diets containing increasing levels of biological silage from tambaqui by-products

Factors^1^	Variables^2^					
	TPT	TRI	GLU	CHO	ALB	UAC
Age						
23 weeks	3.88 ± 1.09^b^	550.34 ± 285.70^b^	225.55 ± 34.84^a^	49.78 ± 26.90^b^	3.18 ± 1.07^b^	2.57 ± 1.77^a^
83 weeks	4.80 ± 1.43^a^	682.31 ± 287.26^a^	181.22 ± 41.56^b^	74.60 ± 29.82^a^	4.83 ± 0.90^a^	1.93 ± 1.33^b^
BST						
0	4.26 ± 1.04^b^	559.69 ± 305.92	192.70 ± 59.10	78.33 ± 43.57^a^	3.77 ± 1.22^c^	1.80 ± 0.78
1	4.76 ± 1.38^a^	510.46 ± 431.14	214.25 ± 47.70	71.55 ± 38.37^a^	3.97 ± 1.20^b^	2.46 ± 1.16
2	4.91 ± 1.46^a^	528.11 ± 344.47	193.95 ± 28.58	60.93 ± 39.39^b^	4.03 ± 1.80^ab^	1.75 ± 1.07
3	4.06 ± 1.55^b^	558.89 ± 398.69	211.95 ± 35.15	50.50 ± 24.70^c^	4.18 ± 1.21^a^	2.69 ± 1.04
4	3.71 ± 0.95^c^	524.46 ± 222.90	204.07 ± 45.01	49.64 ± 21.34^c^	4.06 ± 0.98^a^	2.52 ± 1.06
Effect	p-value					
Age^3^	< 0.01	0.04	< 0.01	< 0.01	< 0.01	0.05
BST^3^	0.04	0.12	0.35	0.05	0.05	0.32
Interaction^4^	0.78	0.15	0.14	0.55	0.71	0.95
CV^5^	10.95	14.72	11.71	7.24	13.22	7.08

^1^ Age = Age of the hens at the beginning of the study. BST = Level of biological silage from tambaqui by-products in the diets

^2^ TPT = Total proteins (g/dL). TRI = Triglycerides (mg/dL). GLU = Glucose (mg/dL). CHO = Total Cholesterol (mg/dL). ALB = Albumin (mg/dL). UAC = Uric acid (mg/dL)

^3^ Means followed by lowercase letters in the column indicate a significant effect of the evaluated factor on the analyzed variable according to Tukey’s test at 0.05

^4^ A p-value above 0.05 demonstrates the direct influence of one factor on the result of the other and vice versa

^5^ CV = Coefficient of variation

### Physical quality of the eggs

**Fig. 3:**
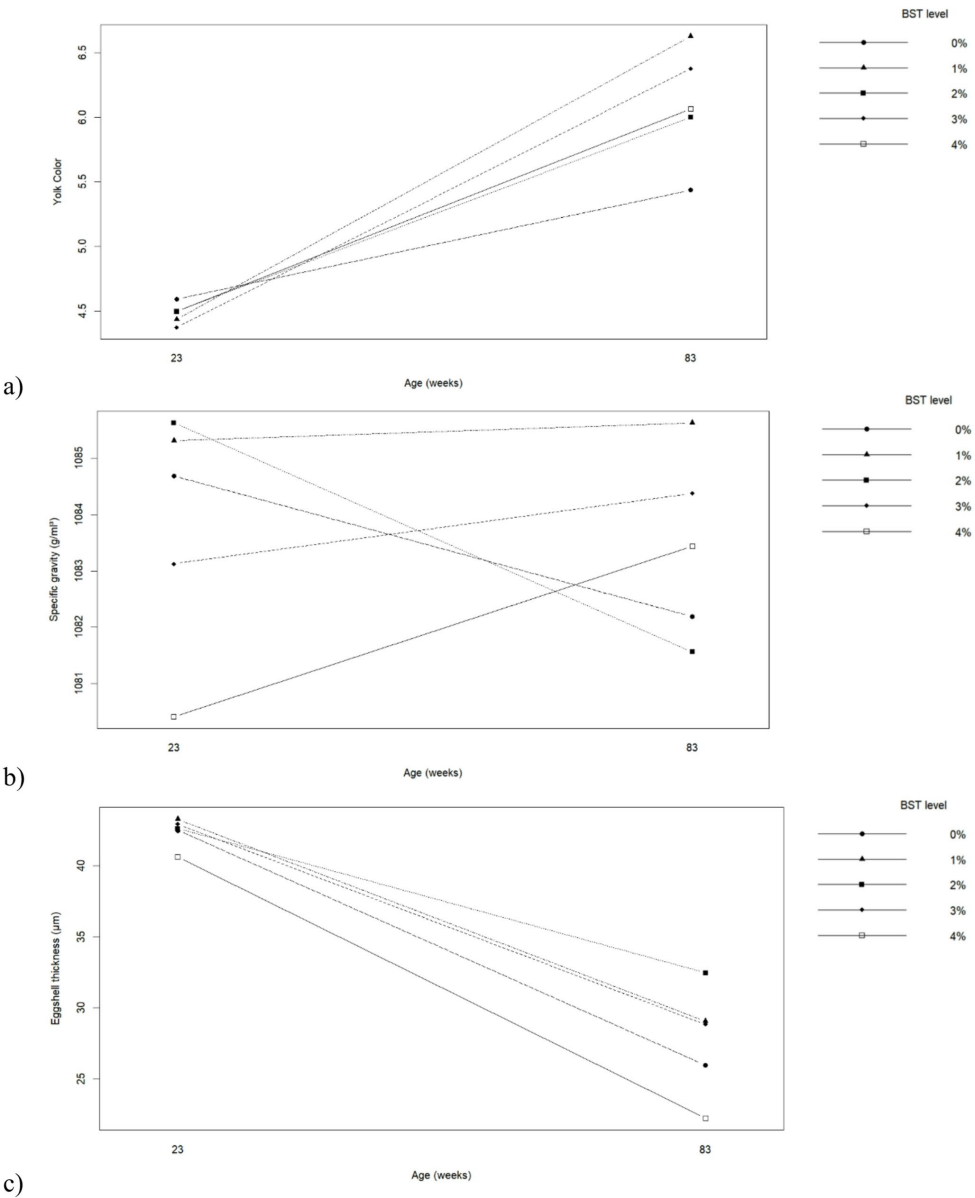
Interaction between biological silage from tambaqui by-products levels in the diets and hen’s age on (**a**) Yolk color, (**b**) Specific gravity and (**c**) Eggshell thickness of eggs produced by commercial hens

Egg physical quality was significantly affected by hen age and BST inclusion for several parameters (Table 6; Fig. 3). Eggs from older hens were heavier, had higher yolk height and yolk color scores, but thinner shells compared with eggs from younger hens (*p* ≤ 0.05). BST inclusion influenced egg weight, albumen percentage, yolk height, albumen height, specific gravity, eggshell thickness, and Haugh unit (*p* ≤ 0.05). Eggs from hens fed 2% BST exhibited the highest albumen height, eggshell thickness, and Haugh unit values, indicating superior internal and external quality. Conversely, 4% BST reduced egg weight, specific gravity, eggshell thickness, and Haugh unit values (*p* ≤ 0.05). Significant interactions between hen age and BST level were observed for yolk color, specific gravity, and eggshell thickness (*p* ≤ 0.05). As shown in Fig. 3, younger hens responded more positively to moderate BST inclusion, whereas older hens showed greater variability in shell quality parameters as BST levels increased.

**Table 6:** Physical quality of eggs produced by commercial hens with different ages fed diets containing increasing levels of biological silage from tambaqui by-products

Factors^1^	Variables^2^										
	EW	Y	A	E	YH	AH	YD	YC	SG	ET	HU
Age											
23 weeks	50.18 ± 7.82^b^	25.34 ± 2.27	49.59 ± 7.46^b^	25.07 ± 1.26^a^	17.07 ± 1.29^b^	7.86 ± 1.57	41.41 ± 1.64	4.48 ± 0.33^b^	1,083.83 ± 2.38	42.40 ± 2.14^a^	90.32 ± 10.03
83 weeks	52.72 ± 4.20^a^	24.88 ± 1.71	53.45 ± 2.54^a^	21.67 ± 0.66^b^	18.62 ± 0.69^a^	7.77 ± 0.75	41.17 ± 1.11	6.10 ± 0.78^a^	1,083.43 ± 2.83	27.71 ± 4.25^b^	89.99 ± 4.29
BST											
0	50.19 ± 6.66^b^	24.65 ± 1.42	53.02 ± 7.28^b^	22.33 ± 1.03	17.38 ± 1.43^b^	7.66 ± 1.24^b^	40.72 ± 1.22	4.87 ± 0.82	1,083.85 ± 2.79^b^	36.99 ± 8.44^b^	89.60 ± 8.43^c^
1	50.68 ± 6.38^b^	24.72 ± 1.90	52.81 ± 8.42^c^	22.47 ± 0.75	17.54 ± 1.14^b^	7.79 ± 1.08^b^	41.30 ± 0.96	5.16 ± 1.10	1,085.41 ± 1.44^a^	38.54 ± 7.33^b^	90.38 ± 6.93^b^
2	51.79 ± 6.66^b^	25.35 ± 2.29	53.49 ± 8.26^b^	21.16 ± 1.06	18.28 ± 1.70^a^	8.44 ± 2.02^a^	41.93 ± 1.39	5.00 ± 0.76	1,084.27 ± 2.68^ab^	39.23 ± 5.25^a^	92.52 ± 9.68^a^
3	53.79 ± 8.09^a^	24.63 ± 2.33	54.42 ± 9.38^a^	20.95 ± 1.10	17.55 ± 1.26^b^	7.68 ± 0.88^b^	41.01 ± 2.14	5.04 ± 1.04	1,083.54 ± 2.31^b^	38.25 ± 7.42^b^	89.01 ± 6.72^c^
4	48.68 ± 6.69^c^	26.60 ± 2.05	52.31 ± 8.26^c^	21.09 ± 1.22	17.18 ± 1.02^b^	7.58 ± 1.27^b^	41.71 ± 1.32	5.02 ± 0.99	1,081.42 ± 1.58^c^	34.49 ± 9.25^c^	89.54 ± 8.93^c^
Effect	p-value										
Age^3^	0.04	0.11	0.05	0.05	0.05	0.57	0.27	< 0.01	0.45	< 0.01	0.89
BST^3^	0.05	0.41	< 0.01	0.38	< 0.01	0.05	0.55	0.72	< 0.01	< 0.01	0.05
Interaction^4^	0.61	0.73	0.94	0.82	0.97	0.81	0.54	0.03	< 0.01	< 0.01	0.91
CV^5^	13.53	8.34	15.21	11.95	7.64	7.22	3.59	8.50	3.23	10.23	9.44

^1^ Age = Age of the hens at the beginning of the study. BST = Level of biological silage from tambaqui by-products in the diets

^2^ EW = Egg weight (g). Y = Yolk (%). A = Albumen (%). E = Eggshell (%). YH = Yolk height (mm). AH = Albumen height (mm). YD = Yolk diameter (mm). YC = Yolk color (ROCHE© scale ranging from 1 to 15). SG = Specific gravity (g/ml³). ET = Eggshell thickness (µm). HU = Haugh unit

^3^ Means followed by lowercase letters in the column indicate a significant effect of the evaluated factor on the analyzed variable according to Tukey’s test at 0.05

^4^ A p-value above 0.05 demonstrates the direct influence of one factor on the result of the other and vice versa

^5^ CV = Coefficient of variation

### Chemical composition of the eggs

**Fig. 4:**
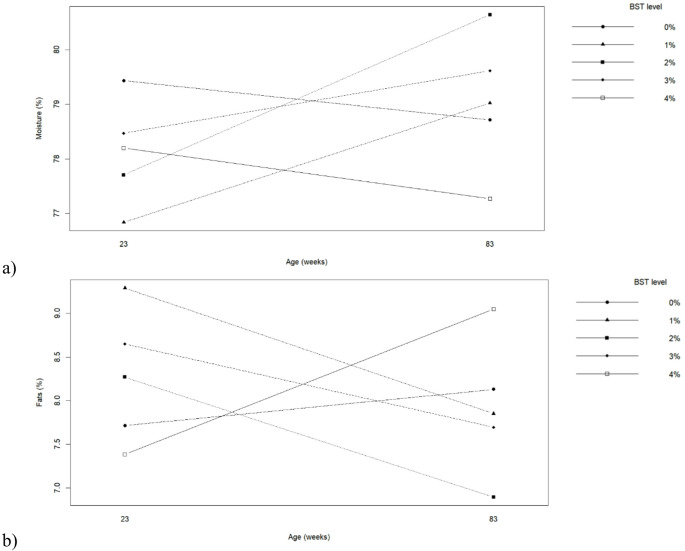
Interaction between biological silage from tambaqui by-products levels in the diets and hen’s age on percentages of (**a**) Moisture and (**b**) Total Fats of eggs produced by commercial hens

Egg chemical composition was influenced by hen age and BST inclusion (Table 7; Fig. 4). Eggs from older hens presented higher moisture content and lower mineral and fat contents compared with those from younger hens (*p* ≤ 0.05). BST inclusion significantly affected moisture and fat contents (*p* ≤ 0.05). Diets containing 1% and 3–4% BST increased yolk fat content, whereas 2% BST resulted in lower fat levels. Egg protein content was not significantly affected by BST inclusion. A significant interaction between hen age and BST level was observed for moisture and fat contents (*p* ≤ 0.05). Figure 4 shows that moderate BST inclusion altered egg composition differently depending on hen age, with younger hens exhibiting more stable compositional responses.

**Table 7:** Proximate composition of eggs produced by commercial hens with different ages fed diets containing increasing levels of biological silage from tambaqui by-products

Factors^1^	Variables^2^			
	MOS	MIN	FAT	PRO
Age				
23 weeks	78.12 ± 1.28^b^	0.80 ± 0.04^a^	8.26 ± 1.10^a^	12.82 ± 1.04
83 weeks	79.05 ± 1.40^a^	0.72 ± 0.05^b^	7.92 ± 0.77^b^	12.31 ± 0.67
BST				
0	79.07 ± 0.47^a^	0.75 ± 0.02	7.92 ± 0.29^b^	12.26 ± 0.25
1	77.92 ± 1.45^b^	0.79 ± 0.06	8.56 ± 0.86^a^	12.73 ± 0.59
2	79.17 ± 2.04^a^	0.74 ± 0.09	7.58 ± 1.06^b^	12.51 ± 1.30
3	79.04 ± 1.35^a^	0.74 ± 0.07	8.17 ± 0.69^a^	12.05 ± 0.74
4	77.73 ± 0.88^b^	0.80 ± 0.04	8.21 ± 1.48^a^	13.26 ± 0.98
Effect	p-value			
Age^3^	0.02	< 0.01	0.05	0.15
BST^3^	0.05	0.10	0.05	0.11
Interaction^4^	0.01	0.12	< 0.01	0.45
CV^5^	1.79	8.60	11.81	7.16

^1^ Age = Age of the hens at the beginning of the study. BST = Level of biological silage from tambaqui by-products in the diets

^2^ MOS = Moisture (%). MIN = Total Minerals (%). FAT = Total Fats (%). PRO = Total Proteins (%). Values are expressed on an as fresh basis

^3^ Means followed by lowercase letters in the column indicate a significant effect of the evaluated factor on the analyzed variable according to Tukey’s test at 0.05

^4^ A p-value above 0.05 demonstrates the direct influence of one factor on the result of the other and vice versa

^5^ CV = Coefficient of variation

### Sensory characteristics of the eggs

**Fig. 5:**
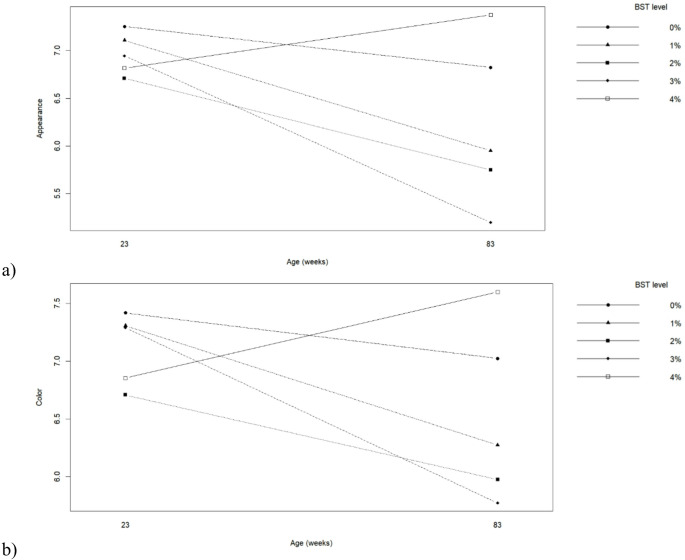
Interaction between biological silage from tambaqui by-products levels in the diets and hen’s age on (**a**) Appearance and (**b**) Color of eggs produced by commercial hens

Sensory evaluation revealed significant effects of hen age and BST inclusion on most attributes (Table 8; Fig. 5). Eggs from younger hens received higher scores for appearance, acidity, color, and taste compared with those from older hens (*p* ≤ 0.05), while aroma was not affected by age. BST inclusion significantly influenced appearance, acidity, color, and taste (*p* ≤ 0.05). Eggs from hens fed 0% and 1% BST obtained the highest acceptability scores. Increasing BST inclusion levels led to a progressive reduction in taste and color scores, particularly at 2% and 3% BST. Eggs from hens fed 4% BST showed higher appearance and color scores compared to intermediate inclusion levels, while taste acceptability remained lower than the control treatment. Significant interactions between hen age and BST level were observed for appearance and color (*p* ≤ 0.05), as illustrated in Fig. 5, indicating differential sensory perception depending on both dietary treatment and hen age.

**Table 8:** Sensory characteristics of eggs produced by commercial hens with different ages fed diets containing increasing levels of biological silage from tambaqui by-products

Factors^1^	Variables^2^				
	APE	ACI	ARO	COL	TAS
Age					
23 weeks	6.96 ± 1.76^a^	7.29 ± 1.68^a^	6.63 ± 1.81	7.11 ± 1.74^a^	7.37 ± 1.59^a^
83 weeks	6.22 ± 2.07^b^	6.57 ± 1.52^b^	6.51 ± 1.76	6.53 ± 1.90^b^	6.72 ± 1.80^b^
BST					
0	7.07 ± 1.51^a^	7.17 ± 1.47^a^	6.63 ± 1.85	7.25 ± 1.65^a^	7.47 ± 1.50^a^
1	6.62 ± 1.79^b^	7.18 ± 1.48^a^	6.46 ± 1.86	6.87 ± 1.72^b^	7.24 ± 1.52^a^
2	6.30 ± 2.11^c^	6.96 ± 1.62^b^	6.49 ± 1.77	6.40 ± 2.01^c^	6.87 ± 1.89^c^
3	6.21 ± 2.13^c^	6.68 ± 1.88^c^	6.56 ± 1.76	6.65 ± 1.87^c^	6.82 ± 1.88^c^
4	7.05 ± 1.90^a^	6.94 ± 1.77^b^	6.75 ± 1.79	7.16 ± 1.80^a^	7.09 ± 1.68^b^
Effect	p-value				
Age^3^	< 0.01	< 0.01	0.48	< 0.01	< 0.01
BST^3^	< 0.01	0.05	0.80	< 0.01	0.04
Interaction^4^	< 0.01	0.59	0.59	< 0.01	0.70
CV^5^	9.09	13.70	17.44	16.76	14.17

^1^ Age = Age of the hens at the beginning of the study. BST = Level of biological silage from tambaqui by-products in the diets

^2^ APE = Appearance. ACI = Acidity. ARO = Aroma. COL = Color. TAS = Taste. Values are expressed on a nine-point hedonic scale (1 = “disliked extremely”; 9 = “liked extremely”)

^3^ Means followed by lowercase letters in the column indicate a significant effect of the evaluated factor on the analyzed variable according to Tukey’s test at 0.05

^4^ A p-value above 0.05 demonstrates the direct influence of one factor on the result of the other and vice versa

^5^ CV = Coefficient of variation

## Discussion

The physicochemical and sensory changes observed in BST throughout fermentation reflect the biochemical and microbiological transformations of the organic matrix. The progressive shift in coloration, from brown to reddish-pink and ultimately to dark brown, is associated with protein degradation, metabolite formation, oxidative degradation of carotenoids, and the formation of melanoidins via Maillard reactions, which are characteristic of fermentative processes involving interactions between proteins and carbohydrates under controlled pH and temperature conditions (Starowicz and Zieliński 2019; Neira et al. 2024; Guimarães et al. 2025). Concurrently, the attenuation of the strong fish odor and the emergence of a slightly acidic or fruity aroma are attributed to the metabolic activity of fermentative microorganisms, particularly lactic acid bacteria, which convert soluble carbohydrates and peptides into organic acids and volatile compounds, thereby contributing to sensory stabilization and microbiological safety of the silage (Abbasiliasi et al. 2017; Yafetto et al. 2022).

The reduction in pH from near-neutral initial values to approximately 5.95 at the end of the fermentation period, together with the marked increase in titratable acidity to about 10.13%, indicates an efficient biological fermentation process and effective acidification of the substrate. Although the final pH remained slightly higher than values typically reported for chemically acidified silages, the elevated titratable acidity reflects substantial accumulation of organic acids. This accumulation plays a decisive role in suppressing spoilage and pathogenic microorganisms, stabilizing the silage matrix, and preserving nutritional quality (Yang et al. 2020; Liu et al. 2022; Rashid et al. 2022).

This apparent dissociation between pH and titratable acidity is well documented in protein-rich, biologically fermented substrates and is largely explained by the buffering capacity of peptides and amino acids derived from fish by-products. These compounds can moderate pH decline while maintaining microbiological safety through acid accumulation (Guimarães et al. 2019; Libonatti et al. 2023).

From a nutritional standpoint, this fermentative profile indicates that BST reached a level of biochemical stabilization compatible with its safe inclusion in poultry diets. It favors protein preservation and limits excessive acidification that could impair palatability. In addition, it supports the generation of fermentation-derived peptides and free amino acids, which may enhance digestibility and nutrient utilization, particularly at low to moderate dietary inclusion levels (Batalha et al. 2019; Guimarães et al. 2021, 2025).

Regarding performance results, the improvement in productive performance observed with the inclusion of BST at low to moderate levels (notably 1–2%) can plausibly be attributed to the nutritional density and biochemical characteristics of the fermented tambaqui by-product. After drying, BST exhibited concentrated crude protein and lipid contents, and when included at moderate levels, it likely enhanced nutrient utilization efficiency without markedly compromising diet formulation (Gauthankar et al. 2021; Guimarães et al. 2021, 2025).

The literature indicates that small inclusions of highly digestible animal-derived ingredients can improve amino acid supply and provide peptides released during the ensiling process (via microbial and enzymatic proteolysis), which may support intestinal function and protein accretion. These effects favor egg production and egg mass while simultaneously improving feed conversion efficiency (Cherian and Quezada 2016; Sakomura and Rostagno 2016; Bezerra and Fonseca 2023; Van Eck et al. 2023). This interpretation is consistent with the present findings, in which the inclusion of 1–2% BST sustained higher egg production and egg mass, along with improved feed efficiency, compared with the control diet.

Conversely, the reduction in performance results at higher BST inclusion levels is consistent with palatability constraints and/or limitations in metabolic processing that may arise with increasing inclusion of animal-derived products (Malenica et al. 2023). Although the silage was microbiologically safe and organoleptically stabilized, it retained the acidic profile characteristic of fermentation and a relatively high lipid fraction after drying (Brelaz et al. 2021; Gauthankar et al. 2021; Libonatti et al. 2023; Guimarães et al. 2025). At higher dietary inclusion levels, these characteristics may reduce voluntary feed intake, as observed at 4% BST, and impair egg production and egg mass. However, this interpretation should be considered with caution, since no direct measurements of palatability, feed preference, or acceptability were conducted in the present study, representing a limitation that precludes definitive conclusions regarding sensory-driven intake responses.

In addition to potential palatability effects, alternative nutritional and physiological mechanisms may explain the reductions in feed intake and performance at higher BST inclusion levels. The increased dietary acid load from fermented ingredients may alter gastrointestinal pH, enzyme activity, and nutrient absorption efficiency (Yang et al. 2020; Liu et al. 2022; Rashid et al. 2022). Moreover, the higher lipid content of BST may enhance susceptibility to lipid oxidation, especially under tropical conditions, generating compounds that impair intake and metabolic efficiency (Brelaz et al. 2021; Van Eck et al. 2023). At the same time, partial replacement of conventional ingredients may lead to amino acid imbalances, limiting protein deposition and egg production (Sakomura and Rostagno 2016; Bezerra and Fonseca 2023). Additionally, fermentation-derived metabolites, beneficial at low levels, may exert adverse effects when excessive. Together, these factors indicate that the decline in performance at higher BST levels is multifactorial, involving digestive, metabolic, and biochemical constraints beyond palatability alone.

In addition, higher inclusion rates of fish-derived ingredients may introduce greater loads of oxidizable lipids or specific volatile compounds, which may not uniformly suppress feed intake across treatments but can nonetheless impair net nutrient availability and productive nutrient partitioning. This effect may be exacerbated under tropical environmental conditions, where feed intake regulation is already sensitive (Batalha et al. 2017, 2018; Guimarães et al. 2019, 2025). These results suggest that the observed negative response was driven by reduced feed intake and/or metabolic efficiency at higher BST inclusion levels, rather than by dietary energy density alone, given that all diets were formulated to be isocaloric (2,850 and 2,900 kcal/kg, respectively), as described in the Materials and Methods section. (Batalha et al. 2017; Guimarães et al. 2019).

The significant interaction between hen age and BST inclusion for feed intake, egg production, and egg mass suggests that older hens were less capable of tolerating higher BST concentrations. This finding is biologically plausible, given age-related differences in digestive function, absorptive capacity, and nutrient partitioning toward egg formation (Susanti et al. 2022; Yenilmez and Atay 2023). In the present study, older hens exhibited lower baseline production and poorer feed efficiency than younger hens, and the decline in performance at higher BST levels was more pronounced. One possible explanation is that, while younger hens may effectively exploit the enhanced nutritional profile of BST at low inclusion levels, older hens may be more sensitive to incremental reductions in palatability or digestibility and to subtle shifts in nutrient balance, which more rapidly translate into reduced egg output (Yenilmez and Atay 2023; Van Eck et al. 2023). This interaction aligns with the premise that age modulates the metabolic utilization of dietary ingredients and helps explain why an inclusion threshold around 2% appears to distinguish beneficial from adverse productive responses (Bain et al. 2016; Cherian and Quezada 2016; Bezerra and Fonseca 2023).

Corroborating these findings, the hematological responses observed in the present study indicate that moderate inclusion levels of BST (1–2%) in the diets maintained hematological parameters within the physiological reference ranges established for commercial hens. According to the literature, these ranges are approximately 2.0 to 3.5 × 10⁶ cells/mm³ for erythrocyte counts, 7.0 to 13.0 g/dL for hemoglobin concentrations, and 22 to 35% for hematocrit values, with natural variation depending on age, genetic line, and physiological status (Rezende et al. 2021; Hassan et al. 2023; Sokolenko et al. 2024; Umoren et al. 2024). Conversely, the observation that higher dietary inclusion levels of BST (≥ 3%) led to values outside these reference limits suggests that these changes may be associated with alterations in erythropoietic balance rather than directly indicating a pathological condition, particularly reflected by reductions in hemoglobin concentration and erythrocyte counts (Sokolenko et al. 2024; Umoren et al. 2024).

These hematological responses should be interpreted in conjunction with the plasma biochemical findings. The increase in total plasma protein observed at 1–2% BST suggests improved protein availability and nitrogen metabolism, which may support erythropoiesis through enhanced amino acid supply for hemoglobin synthesis (Kim et al. 2022; Ahiwe et al. 2024). In contrast, at higher inclusion levels, the absence of further increases in plasma protein, combined with reduced feed intake, may indicate limitations in nutrient intake and utilization, which could contribute to reduced erythrocyte production (Umoren et al. 2024). This effect may be exacerbated in older hens due to age-related declines in erythropoietic efficiency, which reduce the capacity for red blood cell renewal. In addition, increased susceptibility to oxidative stress with advancing age may compromise erythrocyte integrity and lifespan (Ahiwe et al. 2024; Umoren et al. 2024). Furthermore, reduced nutrient absorption efficiency may limit the availability of essential substrates for hemoglobin synthesis, intensifying hematological alterations under higher BST inclusion levels (Ahiwe et al. 2024).

In addition, potential imbalances in micronutrient availability, particularly iron and other cofactors involved in hemoglobin synthesis, cannot be excluded due to the partial replacement of conventional feed ingredients, although these were not directly measured in the present study. Likewise, the higher lipid content of BST may increase susceptibility to oxidative stress, which can impair erythrocyte membrane integrity and reduce cell lifespan (Brelaz et al. 2021; Ahiwe et al. 2024; Umoren et al. 2024). The maintenance of these parameters within physiological intervals at moderate BST inclusion levels indicates that such supplementation may support adequate protein and amino acid-derived peptide metabolism, thereby sustaining erythropoiesis and oxygen transport capacity. This response contributes to the preservation of physiological homeostasis while preventing excessive nutritional or oxidative stress (Kim et al. 2022; Ahiwe et al. 2024).

It is well established that the ensiling process promotes protein hydrolysis, generating smaller peptides and free amino acids that can enhance nutrient absorption and utilization, thereby supporting hematopoiesis (Guimarães et al. 2021). In contrast, the reductions in hemoglobin levels and erythrocyte counts observed at higher BST inclusion rates should be interpreted as being associated with combined effects of reduced feed intake, possible nutrient imbalances, and increased oxidative demand, which may increase susceptibility to lipid peroxidation and compromise erythrocyte integrity (Hassan et al. 2023; Sokolenko et al. 2024), rather than a direct causal effect of BST itself, potentially reflecting subclinical physiological adjustments when dietary inclusion exceeds the birds’ adaptive capacity (Brelaz et al. 2021; Hassan et al. 2023; Sokolenko et al. 2024).

Age-related differences in hematological parameters further reinforce this interpretation. Older hens exhibited higher erythrocyte counts and hematocrit values, whereas younger hens showed higher mean corpuscular volume (MCV), mean corpuscular hemoglobin (MCH), and mean corpuscular hemoglobin concentration (MCHC), indicating distinct erythrocyte profiles associated with age-dependent metabolic strategies (Hong et al. 2021; Sokolenko et al. 2024). Younger hens, characterized by higher production rates and metabolic activity, may rely on fewer but larger and more hemoglobin-rich erythrocytes to meet oxygen demands, while older hens may compensate with a greater number of erythrocytes (Hong et al. 2021; Rezende et al. 2021).

The significant interaction between age and BST inclusion observed for several hematological indices, showing that older hens were less resilient to higher BST levels, supports the interpretation that age-related reductions in digestive efficiency, antioxidant capacity, and metabolic flexibility may increase susceptibility to dietary challenges (Hong et al. 2021; Rezende et al. 2021; Osadcha 2023). Consequently, dietary challenges imposed by higher BST inclusion appear to translate more rapidly into hematological alterations in older birds, whereas younger hens maintained homeostasis up to moderate inclusion levels (Rezende et al. 2021; Osadcha 2023).

Plasma biochemical profiles corroborate these hematological findings and provide additional insight into the metabolic consequences of BST inclusion. The increase in total plasma protein levels at 1–2% BST supports the hypothesis that moderate inclusion enhances protein metabolism and nitrogen utilization, likely due to the high biological value of fish-derived proteins and the presence of fermentation-derived peptides (Kim et al. 2022; Ahiwe et al. 2024). Conversely, the progressive reduction in plasma cholesterol with increasing BST inclusion may be related to alterations in lipid metabolism induced by the fatty acid profile of the silage and by bioactive compounds generated during fermentation, which have been associated with hypocholesterolemic effects in poultry (Van Eck et al. 2023; Zou et al. 2025). The elevation of albumin levels at higher BST inclusion rates, despite declines in performance and hematological indices, may reflect a compensatory hepatic response aimed at maintaining oncotic pressure and transport capacity under metabolic challenge (Umoren et al. 2024; Zou et al. 2025). Importantly, the absence of significant interactions between age and BST for biochemical parameters suggests that, unlike hematological traits, plasma metabolites responded primarily and independently to diet and age. Taken together, these results support the interpretation that hematological alterations at higher BST levels are likely early indicators of physiological imbalance associated with nutritional and metabolic constraints, rather than evidence of overt systemic dysfunction (Hassan et al. 2023; Sokolenko et al. 2024).

Regarding the effects on egg quality, the responses observed for physical traits indicated that moderate inclusion of BST, particularly at 2%, positively modulated key indicators of both internal and external egg quality, whereas higher inclusion levels compromised these attributes. Improvements in albumen height, Haugh unit, and eggshell thickness at 2% BST suggest enhanced protein deposition and shell mineralization, which may be associated with improved amino acid availability and mineral supply derived from BST (Batalha et al. 2018; Guimarães et al. 2025). The proteolytic activity inherent to the biological ensiling process releases low-molecular-weight peptides and free amino acids that are more readily absorbed, thereby favoring albumen synthesis and structural integrity (Ramírez et al. 2013; Nascimento 2023). These improvements are also directly associated with the high biological value of proteins present in BST, which provide a more balanced amino acid profile and enhance albumen protein quality, a key determinant of albumen height and Haugh unit (Reis et al. 2025; Wu et al. 2025). Moreover, the bioavailability of minerals such as calcium and phosphorus from fish-derived ingredients may be higher due to their organic matrix, improving shell mineralization efficiency and contributing to increased eggshell thickness (Javadi et al. 2021; Wu et al. 2025).

In addition, the mineral-rich profile of BST, particularly calcium and phosphorus concentrated after drying, may have contributed to improvements in shell thickness and specific gravity when included at moderate levels (Ramírez et al. 2013). In contrast, the reductions in egg weight, shell quality, and Haugh unit observed at 4% BST likely reflect decreased nutrient intake and reduced nutrient allocation to egg formation, which is consistent with the concomitant decline in productive performance observed at this inclusion level (Ramírez et al. 2013; Batalha et al. 2018; Guimarães et al. 2025). Additionally, higher inclusion levels of BST may increase the susceptibility of yolk lipids to oxidation, which can negatively affect albumen stability and internal egg quality, contributing to the observed reductions in Haugh unit at elevated inclusion levels (Javadi et al. 2021; Reis et al. 2025; Wu et al. 2025).

With respect to the influence of hen age on these attributes, the finding that eggs from older hens exhibited greater weight, higher yolk height, and improved yolk color, but thinner shells, is directly associated with the natural aging process. As hens age increase, eggshell quality typically deteriorates due to reduced calcium absorption efficiency and altered shell gland function, despite increases in egg size (Benavides-Reyes et al. 2021). The significant interaction between age and BST inclusion for yolk color, specific gravity, and shell thickness suggests that younger hens were better able to exploit the nutritional benefits of BST, particularly at moderate inclusion levels, whereas older hens showed greater variability and susceptibility to quality losses as BST levels increased (Han et al. 2023; Yenilmez and Atay 2023). This response reinforces the notion that age-related physiological constraints may limit the ability of older hens to adapt to dietary modifications, especially those involving non-conventional ingredients with highly variable physicochemical characteristics (Bain et al. 2016; Cherian and Quezada 2016; Bezerra and Fonseca 2023).

Regarding the chemical composition of the eggs, the increase in yolk lipid content at 1% and at higher BST inclusion levels (3–4%) likely reflects the higher lipid concentration of the silage itself and its contribution of fatty acids to yolk deposition. Conversely, the lower lipid content observed at 2% BST suggests a more efficient balance between dietary lipid supply and metabolic utilization, which is consistent with the superior performance and egg quality recorded at this inclusion level (Blanco et al. 2014; Jian et al. 2021). This balance may also reflect improved oxidative stability at moderate inclusion levels, whereas excessive lipid intake from BST can predispose yolk lipids to peroxidation, impairing overall egg quality (Javadi et al. 2021; Wu et al. 2025). Age-related differences, characterized by higher moisture content and lower lipid and mineral levels in eggs from older hens, are consistent with previously described physiological changes, indicating shifts in nutrient allocation and egg formation dynamics throughout the laying cycle (Han et al. 2023). The interaction between age and BST inclusion for moisture and lipid content further indicates that dietary lipids using BST were metabolized differently depending on the birds’ physiological stage, with younger hens exhibiting more stable compositional responses (Benavides-Reyes et al. 2021; Han et al. 2023).

Finally, the results of the sensory evaluation indicated that BST inclusion influenced egg attributes that are relevant from the consumer’s perspective, particularly those related to taste and visual perception, while aroma remained largely unaffected. Eggs from hens fed diets containing 0% and 1% BST consistently received the highest scores for appearance, color, and taste, suggesting that low inclusion levels do not compromise, and may even preserve, sensory acceptability. The progressive reduction in taste scores with increasing BST inclusion, especially at 2% and 3%, can plausibly be attributed to the incorporation of fish-derived lipids and fermentation-related metabolites into the egg matrix (Ramírez et al. 2013; Batalha et al. 2018; Guimarães et al. 2021, 2025). These compounds, even at low concentrations, may subtly alter taste perception without necessarily affecting aroma, which is consistent with the absence of significant differences in aroma scores among treatments (Brelaz et al. 2019; Guimarães et al. 2021). According to Kralik et al. (2023), this dissociation between taste and aroma is frequently reported in eggs enriched with alternative lipid sources and reflects the high sensitivity of gustatory perception to minor changes in fatty acid composition and lipid oxidation products.

However, it is important to emphasize that the sensory evaluation conducted in the present study should be interpreted as exploratory, since it was based on untrained panelists and did not include assessments of intra-judge repeatability or controls for expectation bias. These methodological aspects limit the robustness and reproducibility of the sensory findings, and therefore caution is required when extrapolating these results to broader consumer acceptance contexts.

The significant effects of hen age and the age × BST interaction on appearance and color further suggest that sensory perception is modulated not only by diet but also by the birds’ physiological stage. Eggs from younger hens were generally preferred, which is consistent with their superior physical quality parameters, such as albumen height and shell integrity, both of which contribute to visual appeal (Şekeroğlu et al. 2024; Tainika et al. 2024). Interestingly, the partial recovery of appearance and color scores at 4% BST, despite lower taste acceptability, may reflect increased yolk pigmentation derived from silage components, possibly due to the presence of carotenoids in BST (Batalha et al. 2018; Guimarães et al. 2025), even in the presence of reduced palatability. This pattern highlights that improvements in visual attributes do not necessarily translate into greater overall acceptability when taste is negatively affected.

Given these limitations, the sensory results should be considered as indicative trends rather than definitive evidence of consumer preference, and their contribution to the overall interpretation of BST inclusion effects should be regarded as complementary to the productive, physiological, and egg quality findings. Thus, although BST can be incorporated into laying hen diets at low inclusion levels without major sensory drawbacks, taste emerges as the limiting sensory factor at higher inclusion rates of fish by-products, as also reported by Brelaz et al. (2019) and Guimarães et al. (2025), reinforcing the need to balance nutritional and sustainability gains with consumer acceptance thresholds.

In conclusion, the present study indicates that the use of BST in commercial hen diets is conditionally viable, with outcomes strongly dependent on both inclusion level and the physiological age of the birds. Moderate inclusion levels (≤ 2%) were associated with improved feed efficiency, maintenance of egg production, and enhanced egg physical quality, while preserving hematological and plasma biochemical parameters within physiological ranges, particularly in younger hens, which exhibited greater metabolic adaptability. In contrast, higher inclusion levels (≥ 3%) were associated with reductions in feed intake and productive performance, as well as alterations in erythropoietic indicators and increased variability in egg quality traits, especially in older hens, suggesting limitations in digestive efficiency and nutrient utilization under these conditions. Sensory responses indicated that low inclusion levels did not adversely affect consumer-related attributes; however, these findings should be interpreted with caution due to the exploratory nature of the sensory evaluation. From a sustainability perspective, BST represents a promising alternative to partially reduce reliance on conventional ingredients such as corn and soybean meal and to promote the valorization of aquaculture by-products within a circular economy framework. Nevertheless, its practical application requires careful adjustment of dietary inclusion levels according to the physiological stage of the hens, in order to balance productive performance, metabolic stability, and animal welfare, while avoiding adverse effects associated with excessive inclusion.

## Data Availability

The data of this study are available from the corresponding author upon reasonable request.
